# High anion gap and albumin-adjusted anion gap are associated with hospital mortality in intensive care unit patients with liver cirrhosis: A retrospective cohort

**DOI:** 10.1097/MD.0000000000047938

**Published:** 2026-03-13

**Authors:** Liu Zhao, Song Xuefeng, Zhang Qiang, Liu Bo

**Affiliations:** a Department of Emergency Medicine, Beijing Youan Hospital, Capital Medical University, Beijing, China; b Department of Gastrointestinal Oncology, Shanxi Province Cancer Hospital/Shanxi Hospital Affiliated to Cancer Hospital, Chinese Academy of Medical Sciences/Cancer Hospital Affiliated to Shanxi Medical University, Taiyuan, China; c Department of Critical Care Medicine, Peking University Third Hospital, Beijing, China.

**Keywords:** albumin-adjusted anion gap, anion gap, liver cirrhosis, mortality

## Abstract

Data of patients with liver cirrhosis (LC) were collected from the Medical Information Mart for Intensive Care III database to explore whether anion gap (AG) and albumin-adjusted AG (AA-AG) values were associated with outcomes in patients with LC. We retrospectively analyzed data of adult patients with LC. Based on the AG and AA-AG level, patients were then divided into groups according to third percentile. Lowess smoothing was first applied to visualize the crude relationship between AG or AA-AG and inhospital mortality. Survival curves were generated with the Kaplan–Meier and compared by log-rank test. Multivariable logistic regression was constructed to quantify the independent effect of elevated or AA-AG on hospital mortality after adjustment multiple confounding factors. Model discrimination was assessed with area under the receiver operating characteristic curve (AUC) and 95% confidence intervals (CI). Lowess Smoothing technique showed that AG and AA-AG were associated with hospital mortality for patients with LC. Crude outcomes and Kaplan–Meier survival curve analysis revealed that hospital survival rates of patients with high AG and AA-AG values were significantly lower (*P* < .001) compared to those with lower values. After adjusting for multiple confounding factors, analysis revealed that elevated AG (>19 mmol/L) was an independent risk factor for increased inhospital mortality in patients with LC (odds ratio: 1.887 [95% CI: 1.208–2.95]; *P* < .05), and elevated AA-AG (>21.5 mmol/L) was an independent risk factor for increased inhospital mortality in patients with LC (odds ratio: 1.892 [95% CI: 1.229–2.912]; *P* < .05). Specifically, the AG demonstrated an AUC of 0.6704 (95% CI: 0.63–0.71) in predicting hospital mortality. The Model for End-Stage Liver Disease (MELD), on the other hand, exhibited a higher predictive accuracy with an AUC of 0.7186 (95% CI: 0.68–0.76). When AG and MELD were combined, the predictive performance further improved, yielding an AUC of 0.7302 (95% CI: 0.69–0.77). Similarly, the AA-AG showed an AUC of 0.684 (95% CI: 0.64–0.73) in predicting hospital mortality, and when combined with the MELD, the AUC increased to 0.7376 (95% CI: 0.70–0.78). Elevated serum AG (≥19 mmol/L) and AA-AG (≥21.5 mmol/L) were risk factors for inhospital mortality among critically ill patients with LC.

## 1. Introduction

Liver cirrhosis (LC) remains a significant challenge in clinical practice. Liver cirrhosis is characterized by fibrosis of scar tissue and transformation of the normal liver structure into abnormal nodules due to the long-term inflammatory process(es) of chronic liver disease.^[1]^ Cirrhosis and other chronic liver diseases rank 14th among the leading causes of death globally, and have a substantial impact on mortality.^[2,3]^ In recent years, LC has been recognized as a disease not only limited to the liver but a multisystem disease caused by inflammation. Inflammation plays an important role in the development of conditions including hepatorenal syndrome, cirrhotic cardiomyopathy, hepatopulmonary syndrome, and hepatic encephalopathy.^[4]^ In 2017, LC contributed to 1.32 million deaths worldwide, accounting for 2.4% of global mortality.^[5]^ Because LC is a major global health burden, urgent measures are needed to mitigate its impact on morbidity and mortality rates.^[6]^

The anion gap (AG) has long been recognized as a reliable clinical parameter for assessing disturbances in acid–base homeostasis and evaluating disease severity. However, given that AG calculations do not account for variations in serum albumin – a key unmeasured anion – its accuracy in reflecting the true presence of unmeasured anions may be limited. To address this limitation, the albumin-adjusted anion gap (AA-AG) was employed in this study. Albumin-adjusted anion gap is derived by adjusting the conventional AG value based on serum albumin concentrations, thereby providing a more precise estimation of unmeasured anions and enhancing the diagnostic utility of acid–base assessments. In the prediction model of inhospital mortality in patients with LC and sepsis, it was found that AG is a risk factor for inhospital mortality.^[7]^ In addition, the column chart model also found that AG, bilirubin, sodium, and prothrombin time are important prognostic factors for inhospital mortality in patients with esophageal and gastric varices caused by cirrhosis.^[8]^ The serum AG is closely related to the mortality rate of critically ill patients with various diseases.^[9]^ Anion gap is used in most environments, from outpatient monitoring to inpatient intensive care units (ICUs). Supported by decades of experience, the value of the original AG stems from its simplicity.^[10]^ The AG level can be calculated from the electrolyte in the blood through laboratory testing. It is a cheap and effective biochemical indicator. Acid–base imbalance is also a common complication in critically ill patients with LC. The latest research has found that AG was associated with inhospital mortality in critically ill patients with liver failure and shows a moderate predictive value, comparable to the predictive ability of Model for End-Stage Liver Disease (MELD) score.^[11]^ Pan et al found that elevated AA-AG had a significant association with 28-day mortality.^[12]^ Xu et al showed a meaningful link to mortality rates among critically ill patients with cirrhosis.^[13]^ Patients with LC often have hypoalbuminemia due to impaired liver function. However, the effect of serum AG level on outcomes in critically ill patients with LC have been poorly explored. Therefore, the present study aimed to clarify the correlations of AG and albumin-adjusted AG (AA-AG) with hospital mortality in these individuals.

## 2. Materials and methods

### 2.1. Data resources

The Medical Information Mart for Intensive Care (MIMIC-III) database, funded by the National Institutes of Health in the United States,^[14]^ contains information on 46,520 critically ill patients from 2001 to 2012. The MIMIC database was approved by the institutional review boards of the Beth Israel Deaconess Medical Center (2001-P-001699/14) and the Massachusetts Institute of Technology (No. 0403000206), which waived the requirement for individual patient consent because the datasets contained deidentified information. Members of this research team have obtained research permission for the MIMIC database (certificate number: 62208734).

### 2.2. Research object

**Figure 1.**
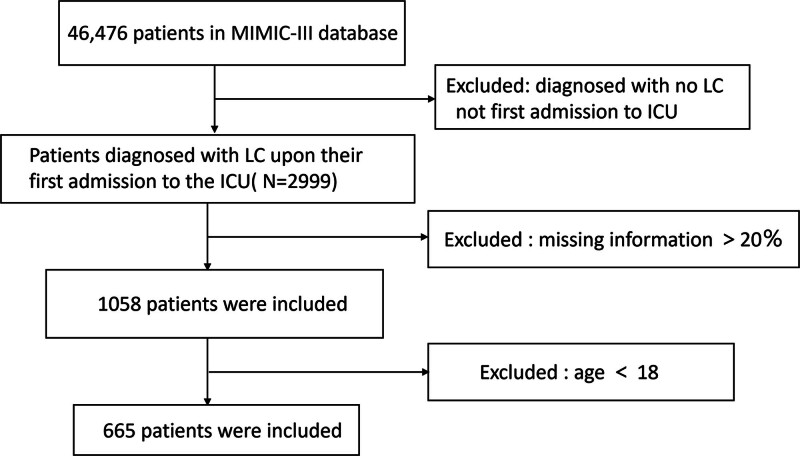
Flow chart of included patients of the study. ICU = intensive care unit, LC = liver cirrhosis, MIMIC-III = Medical Information Mart for Intensive Care.

This study only used data from each patient’s first admission to the ICU. Our inclusion criteria were as follows: adults (age ≥ 18 years old) were admitted to ICU with a diagnosis of liver cirrhosis. We excluded patients aged <18 years, with ICU stay <24 hours. The missing >20% of individual data were excluded. The patient inclusion flowchart is presented in detail in Figure 1.

### 2.3. Data extraction and management

The age, sex, complications (hypertension, diabetes), laboratory data: platelet value, bilirubin value, white blood cell value, total bilirubin value, albumin, alanine aminotransferase, aspartate aminotransferase, anion gap, red-cell distribution width, blood urea nitrogen, creatinine, prothrombin time of patients with LC admitted to the ICU for the first time were extracted from the MIMIC-III database. Simultaneously extract and evaluate the Sequential Organ Failure Score (SOFA), Logistic Organ Dysfunction Score (LODS), and Model for End-Stage Liver Disease (MELD) scores. The main outcome measure is hospital mortality. Using Navicat’s PostgreSQL (PremiumSoft CyberTech Ltd, Hong Kong, China) tool to complete data extraction. Albumin-adjusted AG was obtained using the following formula: albumin-adjusted AG = traditional AG + 2.5 × (4 − serum albumin [g/dL]).^[15]^ Missing data (<20%) were addressed using the random forest algorithm, ensuring robust data processing.

### 2.4. Statistical methods

Statistical analysis was conducted using Stata18 software (StataCorp LLC). The Shapiro–Wilk method was used to test the normality of quantitative data. After testing, the distribution of quantitative data was found to be non normal, described as median (quartile) (M [Q1–Q3]). Non parametric tests (Mann–Whitney *U* test or Kruskal–Wallis test) were used for inter group comparisons. Count data is represented by frequency (rate), and intergroup comparisons are compared using Pearson chi square test. We further grouped the study population according to the quartiles of AG and AA-AG: AG ≤ 12 (Q1), 12 < AG ≤ 19 (Q2), AG > 19 (Q3), and AA-AG ≤ 12 (15), 15 < AG ≤ 21.5 (Q2), AA-AG > 21.5 (Q3). Lowess is a nonparametric regression method that fits smooth curves by performing weighted linear fits over localized subsets of the data, thereby illustrating the crude shape of the exposure–outcome relationship without assuming a global functional form. The Lowess Smoothing technique was used to explore the crude relationship between AG (AA-AG) and hospital mortality. Kaplan–Meier survival curve was drawn to analyze the difference in the the cumulative survival rate between patients with AG and AA-AG. The receiver operating characteristic (ROC) curves were used to compare the predictive value of different indicators (AG, AA-AG, and MELD score) for hospital mortality. A stepwise backward elimination method with a significance level of .05 was used to build the final models.

## 3. Results

### 3.1. Basic characteristics between 2 groups of patients

According to the inclusion criteria and exclusion criteria, a total of 665 patients were enrolled in this study, of whom 406 were survivors and 259 died. Table 1 showed that there was no statistically significant difference between the survival group and the death group in terms of gender (*P* = .779), age (*P* = .99), alanine aminotransferase (*P* = .4348), and albumin (*P* = .3419). In addition, the initial serum AG in the death group was higher than that in the survival group ([17 (14–21) vs 14 (12–17); *P* < .001]); the AA-AG in the death group was higher than that in the survival group ([20 (16.78–23.78) vs 17 (14.25–20); *P* < .001]). Among various complications, there was no significant difference in the distribution of coronary heart disease and diabetes complications between the 2 groups (*P* > .05).

**Table 1:** Basic characteristics of the studied patients.

	All	Survivors (406)	Death (259)	*P*-value
Age, yr	54 (48–62)	55.31 ± 11.30	55.32 ± 11.39	.99
Gender (male)		M 259 (63.79%)	M 168 (64.86%)	.779
PLT, ×10^9^/L (M [Q1–Q3])				
ALB, g/L (M [Q1–Q3])	2.89 (2.5–3.3)	2.84 (2.5–3.3)	2.89 (2.4–3.3)	.3419
WBC, ×10^9^/L (M [Q1–Q3])	8.9 (5.8–13.6)	8.0 (5.5–12.3)	10.2 (6.9–17.2)	.001
LOS, d (M [Q1–Q3])	3.83 (1.97–8.94)	3.44 (1.91–7.82)	4.72 (2.43–10.38)	.0058
AG, mmol/L (M [Q1–Q3])	15 (12–19)	14 (12–17)	17 (14–21)	.001
AA-AG, mmol/L (M [Q1–Q3])	18 (15–21.5)	17 (14.25–20)	20 (16.78–23.78)	.001
TBil, mg/dL (M [Q1–Q3])	5.05 (2.6–12.6)	3.8 (2.1–7.85)	8.4 (4.25–20)	.001
ALT, U/L (M [Q1–Q3])	43 (25–109)	40 (25–109)	47 (24–109)	.4348
AST, U/L (M, M [Q1–Q3])	85.5 (48.5–209)	79 (47–190)	102 (53–238)	.0280
MELD (M [Q1–Q3])	5 (3–6)	4 (2–6)	6 (4–8)	.001
SOFA (M [Q1–Q3])	8 (6–11)	7 (5–10)	10 (8–13)	.001
LODS (M [Q1–Q3])	6 (4–8)	5 (3–7)	7 (6–10)	.001
RDW (M [Q1–Q3])	17.4 (15.7–19.5)	17 (15.4–18.9)	18.2 (16.4–20.3)	.001
BUN, mmol/L (M [Q1–Q3])	31 (19–54)	27 (16–45)	44 (24–66)	.001
Cr, mg/dL (M [Q1–Q3])	1.4 (0.8–2.5)	1.2 (0.8–2.1)	1.8 (1.1–3.2)	.001
PT, s (M [Q1–Q3])	19 (16.3–23.1)	18.2 (15.8–21.2)	20.9 (17.8–27.5)	.001
Complication				
Coronary		16 (3.9%)	10 (3.86%)	.959

AA-AG = adjusted anion gap, AG = anion gap, ALB = serum albumin, ALT = alanine aminotransferase, AST = aspartate aminotransferase, BUN = blood urea nitrogen, Cr = serum creatinine, LODS = Logistic Organ Dysfunction Score, LOS = length of hospital stay, MELD = Model for End-Stage Liver Disease, PLT = platelet count, PT = prothrombin time, RDW = red-cell distribution width, SOFA = Sequential Organ Failure Assessment, TBil = total bilirubin, WBC = white blood cell count.

### 3.2. Lowess Smoothing

**Figure 2.**
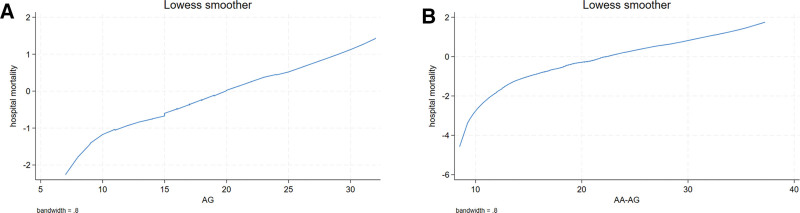
Lowess Smoothing of AG and AA-AG. AA-AG = albumin-adjusted anion gap, AG = anion gap.

Figure 2A shows the relationship between AG and hospital mortality for patients in ICU using the Lowess Smoothing technique. Figure 2B shows the relationship between AA-AG and hospital mortality for patients in ICU using the Lowess Smoothing technique.

### 3.3. Crude outcomes stratified by AG and AA-AG categories

Table 2 shows crude outcomes stratified by AG and AA-AG categories. According to the third percentile of AG, the number of mortality rate of Q1, Q2, and Q3 were 45 (24.32%), 115 (35.17%), and 99 (64.71%) respectively (*P* < .001). The results showed that AG was associated with increased hospital mortality compared with low AG levels for patients (all *P* < .001). According to the third percentile of AA-AG, the number of mortality rate of Q1, Q2, and Q3 were 35 (20.83%), 122 (36.53%), and 102 (62.58%) respectively (*P* < .001). The results showed that AA-AG was associated with increased hospital mortality compared with low AA-AG levels for patients (*P* < .001).

**Table 2:** Unadjusted outcomes by AG categories in patients for mortality.

	AG				AA-AG			
	Q1, N = 185	Q2, N = 327	Q3, N = 153		Q1, N = 168	Q2, N = 334	Q3, N = 163	
Outcomes	AG ≤ 12	12 < AG ≤ 19	AG > 19		AA-AG ≤ 15	15 < AG ≤ 21.5	AA-AG > 21.5	
Hospital mortality, n (%)	45 (24.32%)	115 (35.17%)	99 (64.71%)	*P* < .001	35 (20.83%)	122 (36.53%)	102 (62.58%)	*P* < .001

AA-AG = adjusted anion gap, AG = anion gap.

### 3.4. Survival analysis for the included patients

**Figure 3.**
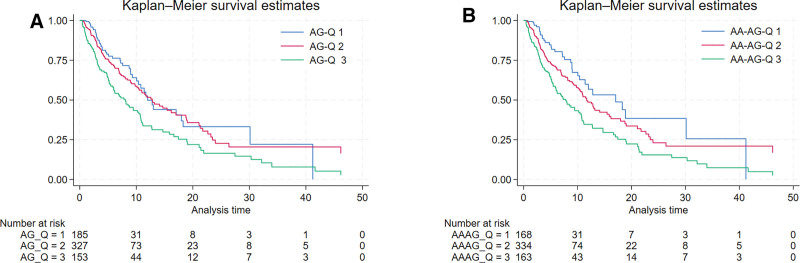
Kaplan–Meier survival curves of AG and AA-AG. AA-AG = albumin-adjusted anion gap, AG = anion gap.

The Kaplan–Meier (K–M) survival curve illustrated that patients with liver cirrhosis elevated levels of AG and AA-AG had decreased survival rates, as shown in Figure 3 (*P* < .0001) in ICU based on (Fig. 2A) AG and AA-AG (Fig. 2B).

### 3.5. Adjusted ORs for hospital mortality using AG and AA-AG

A stepwise backward elimination method with a significance level of .05 was used to build the final models. After adjusting for variables with *P* < .05 from Table 1, Table 3 showed adjusted odds ratios (ORs) for hospital mortality using AG. The results showed that higher AG (Q3) was associated with increased hospital mortality compared with low AG levels (Q1) for patients (OR: 1.887; 95% CI: 1.208–2.95; *P* < .05); and higher AA-AG (Q3) was associated with increased hospital mortality compared with low AA-AG levels (Q1) for patients (OR: 1.892; 95% CI: 1.229–2.912; *P* < .05).

**Table 3:** Adjusted ORs using AG and AA-AG as the design variable in patients for hospital mortality.

	AG				AA-AG			
Variable	OR	95% CI		*P*	OR	95% CI		*P*
Q1	Ref				Ref			
Q3	1.887	1.208	2.950	.005	1.892	1.229	2.912	.004
RDW	1.115	1.042	1.193	.002	1.120	1.046	1.198	.001
WBC	1.005	1.023	1.080	.001	1.048	1.020	1.077	.001
TBil	1.031	1.012	1.050	.001	1.031	1.012	1.050	.001
PT	1.030	1.004	1.057	.02	1.033	1.007	1.059	.013
LODS	1.189	1.105	1.280	.001	1.19	1.106	1.281	.000

AA-AG = adjusted anion gap, AG = anion gap, CI = confidence intervals, LODS = Logistic Organ Dysfunction Score, OR = odds ratio, PT = prothrombin time, RDW = red-cell distribution width, TBil = total bilirubin, WBC = white blood cell count.

### 3.6. ROC curve analysis

**Figure 4.**
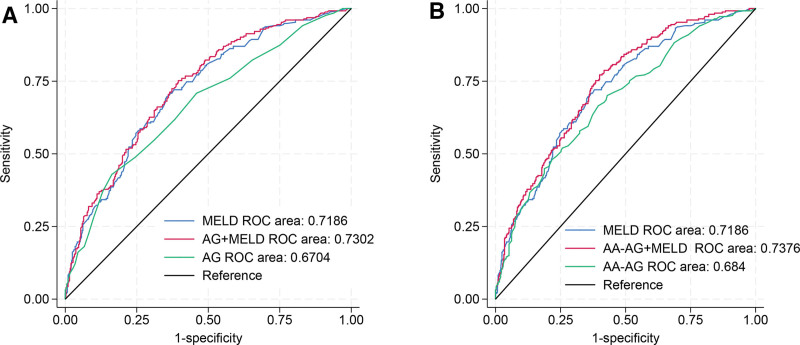
ROC curve analysis of AG and AA-AG predicting inhospital mortality. AA-AG = albumin-adjusted anion gap, AG = anion gap, MELD = Model for End-Stage Liver Disease, ROC = receiver operating characteristic.

The ability of AG to predict hospital mortality was (AUC: 0.6704; 95% CI: 0.63–0.71); ability of MELD to predict hospital mortality was (AUC: 0.7186; 95% CI: 0.68–0.76); AUC was 0.7302 (95% CI: 0.69–0.77) when combined AG and MELD (Fig. 4A). The ability of AA-AG to predict hospital mortality was (AUC: 0.684; 95% CI: 0.64–0.73); AUC was 0.7376 (95% CI: 0.70–0.78) when combined AA-AG and MELD (Fig. 4B). Sensitivity, specificity, AUC, and cutoff levels were shown in Table 4.

**Table 4:** Sensitivity, specificity, AUC, and cutoff levels.

Variable	Sensitivity	Specificity	AUC	95% CI	*P*	Cutoff
AG	61.81%	61.73%	0.6704	0.63–0.71	.0216	16
MELD	66.80%	65.27%	0.7186	0.68–0.76	.0201	29
AG + MELD	67.72%	67.16%	0.7302	0.69–0.77	.0195	44
AA-AG	63.39%	62.72%	0.684	0.64–0.73	.0212	18.5
AA-AG + MELD	67.32%	64.94%	0.7376	0.70–0.78	.0193	47.5

AA-AG = adjusted anion gap, AG = anion gap, AUC = area under the curve, CI = confidence intervals, MELD = Model for End-Stage Liver Disease.

## 4. Discussion

This study revealed the relationship between serum AG levels, AA-AG level and hospital mortality in patients with LC. The results showed that the higher the serum AG and AA-AG levels, the higher the hospital mortality rate. After adjusting for confounding factors, elevated levels of AG and AA-AG were both risk factors for inhospital mortality in critically ill patients with LC.

Elevated AG is an independent risk factor for inhospital mortality among other patients.^[16,17]^ Anion gap is influenced by more indicators, and the most common indicator leading to an increase in AG is metabolic acidosis, which means excessive production of organic acids, such as the accumulation of lactic acid. Some speculate that was related to the hypoxia and shock caused by infection-induced excessive lactate production, as well as the impaired liver lactate clearance function leading to a decrease in lactate clearance rate.^[18]^ With the invention of automated analyzers, electrolyte measurements can be performed on critically ill patients, and serum AG measurements are becoming increasingly easy. Therefore, many studies have revealed the relationship between serum AG levels and clinical outcomes or predicting prognosis in critically ill patients.^[19]^ The hospital and ICU mortality rates of patients with subarachnoid hemorrhage increase with the increase of serum AG concentration.^[20]^ Elevated serum AG levels are an independent, significant, and reliable predictor of all-cause mortality in subarachnoid hemorrhage.^[21]^ Similarly, our research findings show a positive correlation between serum AG and inhospital mortality in critically ill patients with LC.

Although serum AG levels are closely related to the ICU mortality rate of heart failure patients, and the specific mechanism is not yet clear. Research has shown that AG can not only assess the severity of diseases, but also predict the prognosis of patients. AG is not a fixed value and is influenced by various factors such as changes in albumin levels.^[22,23]^ In addition to the kidneys and lungs, the liver is also an important organ for regulating acid–base balance, and liver dysfunction may be accompanied by complex acid–base imbalances.^[24]^ When liver disease progresses to the late stage, the most common sodium imbalance is high-volume hyponatremia.^[25]^ In end-stage liver disease patients, hypokalemia is more common, and the overall potassium level of liver disease patients may decrease by 30% to 40%. Meanwhile, electrolyte disorders in liver failure may vary greatly, depending largely on the severity of the liver disease and the presence or absence of acute kidney injury (AKI). Up to 30% to 50% of hospitalized patients with decompensated cirrhosis may experience AKI.^[26]^ In the absence of AKI, the kidneys compensate by excreting bicarbonate while retaining chloride, ultimately leading to hyperchloremia. The use of diuretics in cases of renal insufficiency may exacerbate hypokalemia.^[11]^

The survival of LC depends not only on the severity of the liver disease, but also on the presence of comorbidities.^[27]^ With the increase of the age of patients with cirrhosis and the prevalence of secondary cirrhosis of nonalcoholic fatty liver, cardiovascular disease, malignant tumor, diabetes, sarcopenia, and weakness are expected to become the main factors leading to adverse results.^[28]^

In future, we need validate the anion-gap cutoff value in a multicenter, prospective cohort comprising hospitalized cirrhotic patients. Anion-gap values will be collected within the first 24 hours of patient admission and correlated with predefined, patient-centered endpoints. Concurrently, acid–base variables (including pH and lactate levels) will be recorded to elucidate the mechanistic role of an elevated anion gap in contributing to mortality.

## 5. Limitations

To our knowledge, this is the first study to show an independent association between AG, AA-AG and hospital mortality in patients with LC admitted to the ICU. However, this study has some limitations. First, due to the MIMIC database, the deletion rate of some variables (such as lactate level, pH, PaO_2_, and PaCO_2_) is close to 35%, and multiple imputation based on 5 replicates is used to reduce bias. Second, the diagnosis of LC is based on administrative codes. Although the first diagnostic sequence was used, some erroneous associations may be caused by misclassification.

## 6. Conclusions

Elevated serum AG (≥19 mmol/L) and AA-AG (≥21.5 mmol/L) are an independent risk factor for hospital mortality in critically ill patients with LC.

## Author contributions

**Conceptualization:** Liu Bo.

**Data curation:** Liu Zhao, Liu Bo.

**Formal analysis:** Song Xuefeng, Zhang Qiang, Liu Bo.

**Investigation:** Song Xuefeng.

**Methodology:** Liu Bo.

**Software:** Zhang Qiang.
